# Modeling time-varying phytoplankton subsidy reveals at-risk species in a Chilean intertidal ecosystem

**DOI:** 10.1038/s41598-024-57108-9

**Published:** 2024-03-24

**Authors:** Casey Duckwall, John L. Largier, Evie A. Wieters, Fernanda S. Valdovinos

**Affiliations:** 1 Department of Environmental Science and Policy, University of California, Davis, Wickson Hall, 1 Shields Avenue, Davis, CA 95616 USA; 2 Graduate Group in Applied Mathematics, University of California, Davis, Mathematical Sciences Building, 1 Shields Avenue, Davis, CA 95616 USA; 3 Bodega Marine Laboratory, University of California, Davis, 2099 Westshore Drive, Bodega Bay, CA 94923 USA; 4 Estación Costera de Investigaciones Marinas and Millennium Nucleus for the Ecology and Conservation of Temperate Mesophotic Reef Ecosystems (NUTME), Faculty of Biological Sciences, Pontificia Universidad Católica de Chile, Osvaldo Marin 1672, Las Cruces, Chile

**Keywords:** Ecology, Ecological modelling, Ecological networks, Population dynamics

## Abstract

The allometric trophic network (ATN) framework for modeling population dynamics has provided numerous insights into ecosystem functioning in recent years. Herein we extend ATN modeling of the intertidal ecosystem off central Chile to include empirical data on pelagic chlorophyll-a concentration. This intertidal community requires subsidy of primary productivity to support its rich ecosystem. Previous work models this subsidy using a constant rate of phytoplankton input to the system. However, data shows pelagic subsidies exhibit highly variable, pulse-like behavior. The primary contribution of our work is incorporating this variable input into ATN modeling to simulate how this ecosystem may respond to pulses of pelagic phytoplankton. Our model results show that: (1) closely related sea snails respond differently to phytoplankton variability, which is explained by the underlying network structure of the food web; (2) increasing the rate of pelagic-intertidal mixing increases fluctuations in species’ biomasses that may increase the risk of local extirpation; (3) predators are the most sensitive species to phytoplankton biomass fluctuations, putting these species at greater risk of extirpation than others. Finally, our work provides a straightforward way to incorporate empirical, time-series data into the ATN framework that will expand this powerful methodology to new applications.

## Introduction

Oceanic subsidies of phytoplankton are essential to the persistence of near-shore intertidal communities^1^. In upwelling systems, increased phytoplankton subsidy increases recruitment, growth, and reproduction of invertebrate populations^2–5^, which increases the secondary productivity transferred to higher trophic levels in intertidal food webs^6–8^. These subsidies, however, are highly variable due to complex dynamics that control phytoplankton blooms in wind-driven upwelling regions. Phytoplankton show variability across several timescales including hours (tidal and diel cycles), days and weeks (synoptic variability in winds), months (intraseasonal and seasonal cycles), and years (interannual cycles)^9–11^. Further, the delivery of pelagic phytoplankton to nearshore/intertidal habitats is controlled by cross-shore exchange processes, typically dominated by wave-driven circulation^12–14^. This complex variability in phytoplankton subsidies forces non-autonomous dynamics (i.e., explicitly dependent on time) into the productivity of intertidal food webs, which challenges the autonomous approach traditionally used to model food web dynamics (i.e., dependent only on fixed parameters representing the local trophic and demographic rates). These non-autonomous factors will most likely cause intertidal food webs to be dominated by transient dynamics^15^, as opposed to equilibrium dynamics, by repeatedly pushing the system away from any trajectory approaching equilibrium. Understanding the effects of these non-autonomous dynamics on ecological systems is an important new frontier in community ecology given the dramatic environmental changes currently altering ecosystem dynamics worldwide.

The Humboldt Current System supports a nutrient-rich and high-diversity ecosystem off the coast of Chile and Peru^16^. These waters are among the most productive marine ecosystems in the world^17,18^ and exhibit dramatic annual fluctuations in phytoplankton abundance due to several, often interacting forces^19^. The high productivity of this system is supported by wind-driven upwelling, a bulk transport process that forces surface water offshore to be replaced with deep, nutrient-rich water^9,20–22^. Upwelling favorable winds exhibit spatiotemporal variability, driving synoptic (shorter-term fluctuations lasting only for days or weeks) and seasonal cycles in upwelling, nutrient concentration, and oceanic phytoplankton density^9,23^. Additionally, global oceanographic and meteorological phenomena, such as the El Niño-Southern Oscillation and the Pacific Decadal Oscillation, alter productivity by introducing environmental variability on time scales of 4–7 and 20–30 years, respectively^24,25^. Finally, wind-driven upwelling is being modified by global climate change^18,26,27^, resulting in significant changes in coastal phytoplankton concentrations^28^; further, pelagic-intertidal coupling can be expected to change with alterations in the winds, waves and stratification that control exchange between the coastal ocean and shoreline habitats. This complex array of interacting processes makes the variability of oceanic phytoplankton subsidizing intertidal communities extremely difficult to predict or model from first principles. Therefore, we propose the use of empirical oceanic phytoplankton time series data to represent the variability of the phytoplankton subsidizing intertidal food webs. Specifically, our present contribution uses a time series of oceanic phytoplankton (measured as chlorophyll-a) from the central coast of Chile to evaluate the effects of such variability on the dynamics of a rocky intertidal food web.

The bioenergetic model^29^ expanded to large food webs by the Allometric Trophic Network (ATN) model^30^ has been successful in capturing the dynamic processes of aquatic food webs, from predicting interaction strengths of rocky-intertidal food webs^31^ to modeling the food web dynamics of lakes^32^. This model uses biomass (energy) as a currency and has been greatly influential toward understanding mechanisms behind community function and stability^33,34^ and for predicting consequences of environmental changes and human exploitation on diversity^6,35–39^. A main reason for its success is that the ATN model leverages several trophic and metabolic processes that scale with the body mass of aquatic organisms^40–43^, allowing researchers to use allometric scaling to parameterize the model for any system where body masses of the interacting species are known. This model, however, only accounts for the autonomous dynamics that emerge from allometrically scaled demographic and interaction rates. Previous work shows how the dynamics of the ATN model applied to the rocky intertidal system we study here are affected by including phytoplankton subsidy at a constant rate (fixed throughout the entire simulation), and subsequently increasing or decreasing this rate based on expectations from different climate change scenarios^6^. Here, we advance the field by modeling these subsidies more realistically by incorporating their high temporal variability, using time-series of chlorophyll-a to incorporate the empirical variability of oceanic phytoplankton into the subsidies received by the rocky intertidal food web.

Our specific objectives are to evaluate how the rocky intertidal food web model of the central coast of Chile responds to (1) the variability of pelagic phytoplankton abundance and (2) the rate of pelagic-intertidal mixing. These two factors combine to determine the flux of particulate food from the ocean to intertidal habitats. We found that while this pelagic subsidy is essential for fueling the intertidal food web, the high level of temporal variability (enhanced by rapid pelagic-nearshore exchange) increases biomass fluctuations putting species at increased risk of extirpation, and that variation in abundance due to phytoplankton subsidy is most pronounced in top predators relative to other types of species.

## Results

**Figure 1:**
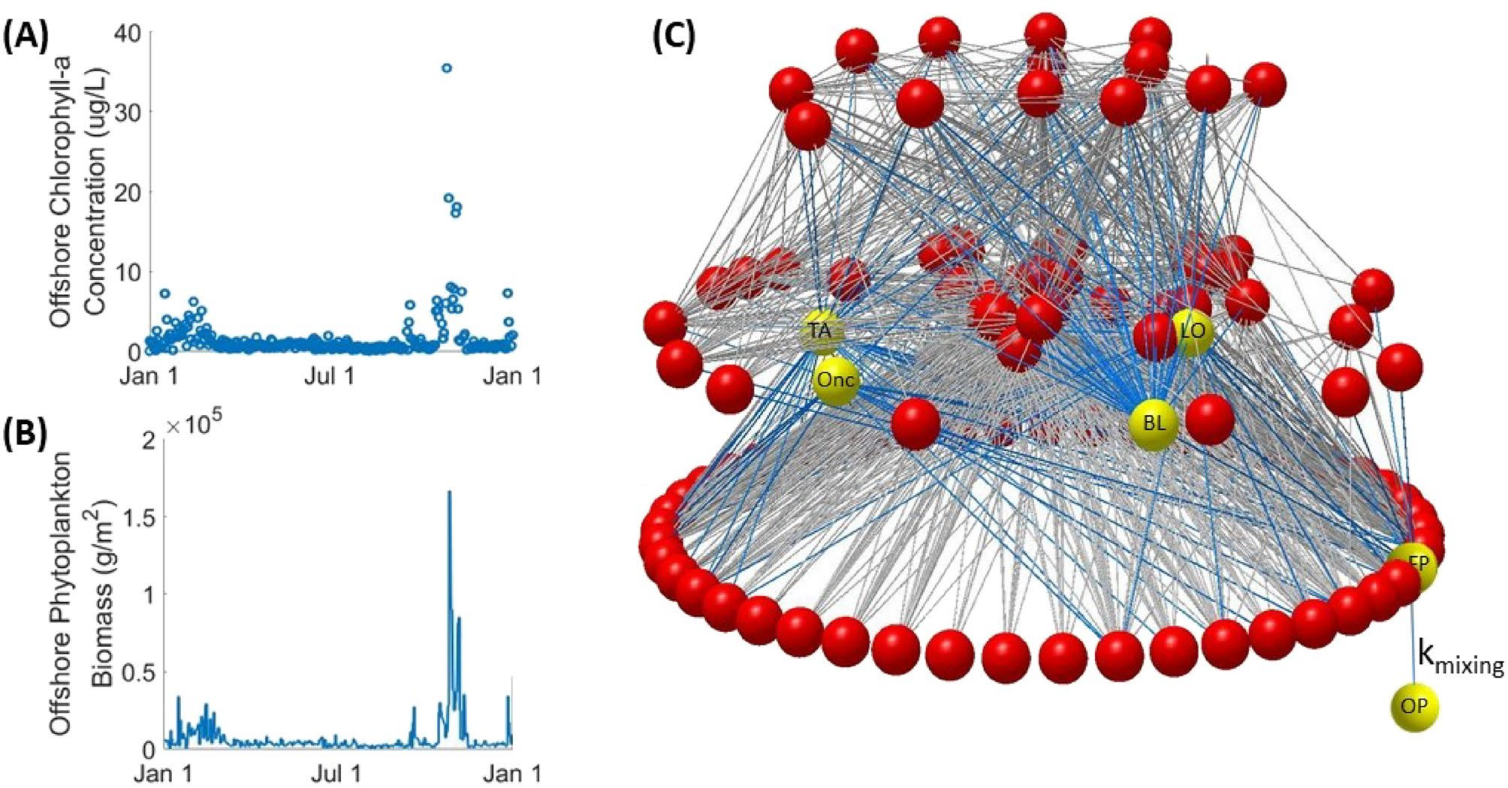
(**A**) Empirical timeseries dataset of offshore chlorophyll-a measurements in 2003. (**B**) Continuous spline fit to scaled empirical dataset. (**C**) Graphical representation of the Las Cruces food web network. Nodes are vertically arranged by using prey-averaged trophic level. Important nodes are labeled as follows: *OP* offshore phytoplankton, *FP* food web phytoplankton, *BL* the barnacle, *B. laevis, Onc* the sea snail, *Onchidella sp.*, *TA* the sea snail, *T. atra*, *LO* the sea snail, *L. orbignyi*.

Figure 1 outlines the study setup. Open-water chlorophyll-a measurements were obtained for the research site near Las Cruces, Chile (Fig. 1A). These empirical values were scaled to model units (biomass per area measured in g/m^2^, displayed as g/m^2^ or kg/m^2^ as denoted), and a spline curve was fit to the data (Fig. 1B) to create a continuous curve of offshore phytoplankton biomass. A network representation of the intertidal ecosystem is provided in Fig. 1C with nodes representing the intertidal species and links representing their associated trophic interactions. The height of a species’ node corresponds to its trophic level. Variable pelagic phytoplankton (offshore phytoplankton, OP) drives this food web network by subsidizing phytoplankton in the intertidal habitat (foodweb phytoplankton, FP). The rate constant, k_mixing_, controls the rate at which pelagic phytoplankton is mixed into the food web network. k_mixing_ is assigned values of 0.1, 1.0, and 10 h^−1^ as denoted. For ease of illustration, we will focus on results from 2003; however, trends are consistent across all years modeled. Results from other years are included in the supplementary materials and referenced where relevant. Summary information for the empirical dataset is provided in Supplementary Table S1.

### Species’ biomasses respond differently to phytoplankton variability depending on their food web connections

We began our investigation into the effects of pulses of high pelagic phytoplankton on intertidal population dynamics using an intermediate rate of pelagic-intertidal mixing, k_mixing_ = 1.0 h^−1^.

**Figure 2:**
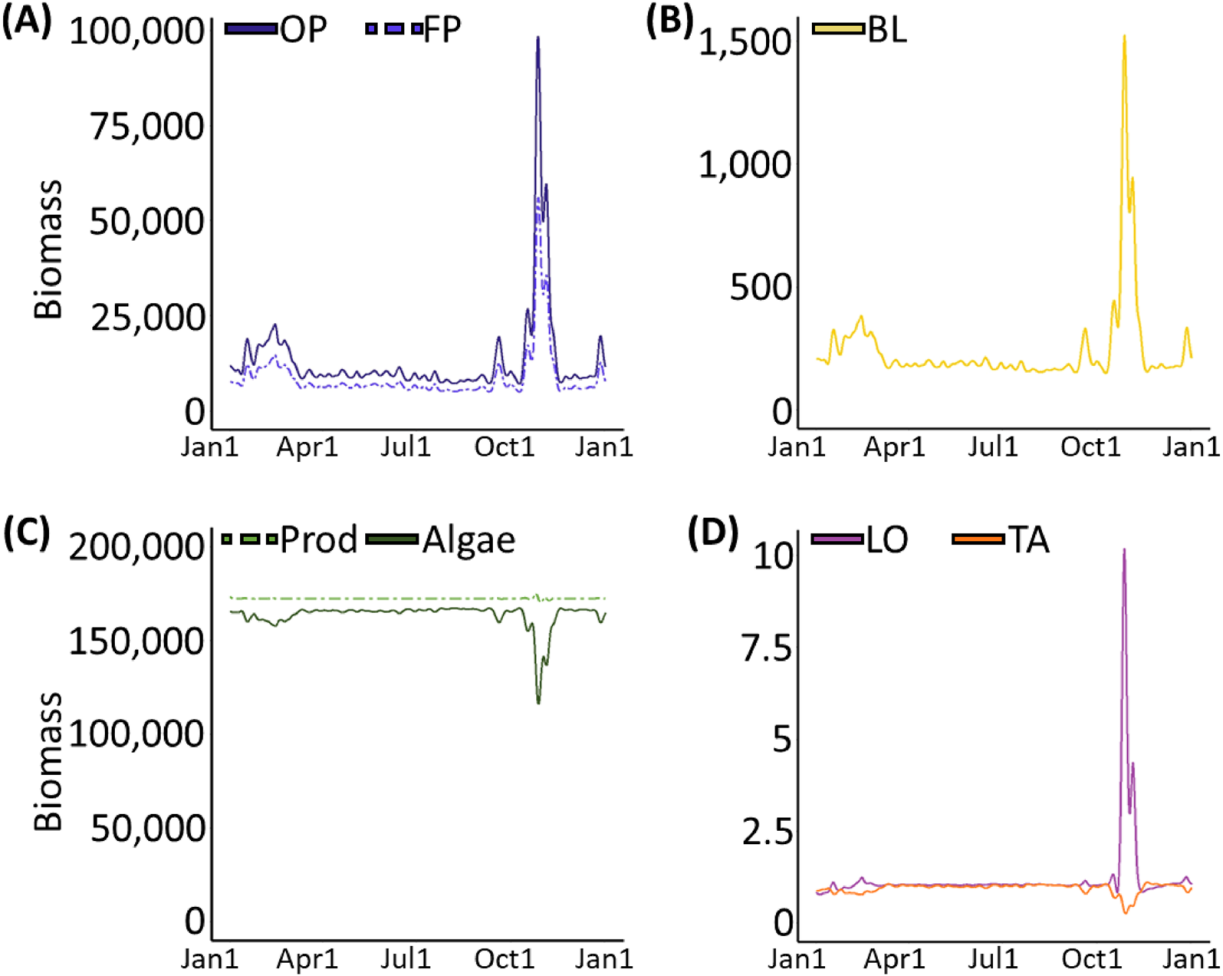
Biomass curves simulated using empirical data from 2003 for (**A**) offshore phytoplankton (OP, solid line) and food web phytoplankton (FP, dashed line), (**B**) the barnacle, *B. laevis* (BL), (**C**) summed biomass of all producer species (Prod, dashed line) and algal species only (Algae, solid line), and (**D**) the omnivorous sea snail, *L. orbignyi* (LO), and the herbivorous sea snail, *T. atra* (TA). Biomasses are shown in g/m^2^. These results use an intermediate pelagic-intertidal mixing rate, k_mixing_ = 1.0 h^−1^.

Pulses of high pelagic phytoplankton, such as the bloom event in late 2003 (Fig. 2A), are characteristic of seasonal upwelling in central Chile^23^. Intermediate to high peaks in offshore phytoplankton were observed in 10 of the 12 years modeled (Table 1, Supplementary Figs. S1, S2). We found that the abundances of all filter feeder species were strongly and positively influenced by increased phytoplankton subsidy (Supplementary Fig. S3). Our model assumes that intertidal particulate organic matter (comprised of phytoplankton and baseline detritus) is the only resource available to filter feeders. Consequently, the biomasses of filter feeder species are tightly coupled with the abundance of intertidal phytoplankton. Figure 2B shows the response of the filter-feeding barnacle, *B. laevis*, an important food source for omnivore sea snails, to the elevation in offshore phytoplankton that occurred in late 2003.

**Table 1:** Results list peak offshore phytoplankton abundance and simulated extirpation status of *Onchidella* under each pelagic-intertidal mixing scenario investigated.

Year	Peak OP abundance	Status under		
		K_mixing_ = 10	K_mixing_ = 1	K_mixing_ = 0.1
1999	297,260	e	e	
2000	191,120	e	e	
2001	47,860	e		
2002	131,400	e	e	
2003	172,440	e	e	
2004	50,338	e		
2005	64,821	e		
2006	74,076	e	e	
2007	179,560	e	e	
2008	61,371	e		
2009	27,577			
2010	17,871			

Cells with an ‘e’ signify simulations in which *Onchidella* experienced extirpation, and empty cells signify simulations where *Onchidella* survived.

The total biomass of all producer species (including phytoplankton) remained relatively constant across the year (labeled Prod in Fig. 2C), but the total biomass of algal species was negatively influenced by increased phytoplankton subsidy (labeled Algae in Fig. 2C). These two results are explained by the community-level carrying capacity that is shared by all producer species of the model (see “Methods” section, Eq. 3). This carrying capacity limits the total biomass of producers such that when a producer species increases its biomass others will decrease theirs due to competition for space, light, or other resources. These results relating phytoplankton subsidy with total producer and non-planktonic producer biomass were consistent across all years modeled (Supplementary Fig. S1).

Responses among consumer species were more varied. For example, different sea snail species exhibited different responses to the Oct.-Nov. 2003 elevation in phytoplankton based on their feeding preference as either omnivore or herbivore (Supplementary Fig. S4). On the one hand, omnivorous sea snails like *L. orbignyi* (LO in Fig. 2D) directly consume filter-feeding barnacles, placing them two nodes above food web phytoplankton. Consequently, our model shows biomass variations in omnivorous sea snails similar to those observed in phytoplankton and their filter feeder prey. On the other hand, herbivorous sea snails like *T. atra* (TA in Fig. 2D) consume algae but not barnacles nor any other consumers of phytoplankton. Consequently, increased phytoplankton affected herbivorous sea snails only indirectly by increasing the abundance of their predators and suppressing the growth of their resources. Through this example, we observe that offshore phytoplankton alters both top-down and bottom-up forces differentially even for related (within class) species, and that these differences can be made evident through inspection of their network connections. This trend—herbivorous sea snails decreasing their biomass in response to elevated offshore and foodweb phytoplankton and omnivorous sea snails increasing their biomass in response to elevated offshore and foodweb phytoplankton—is consistent across all twelve years modeled (Supplementary Fig. S1).

### Increased pelagic-intertidal mixing increases biomass fluctuations putting species at increased risk of extirpation

**Figure 3:**
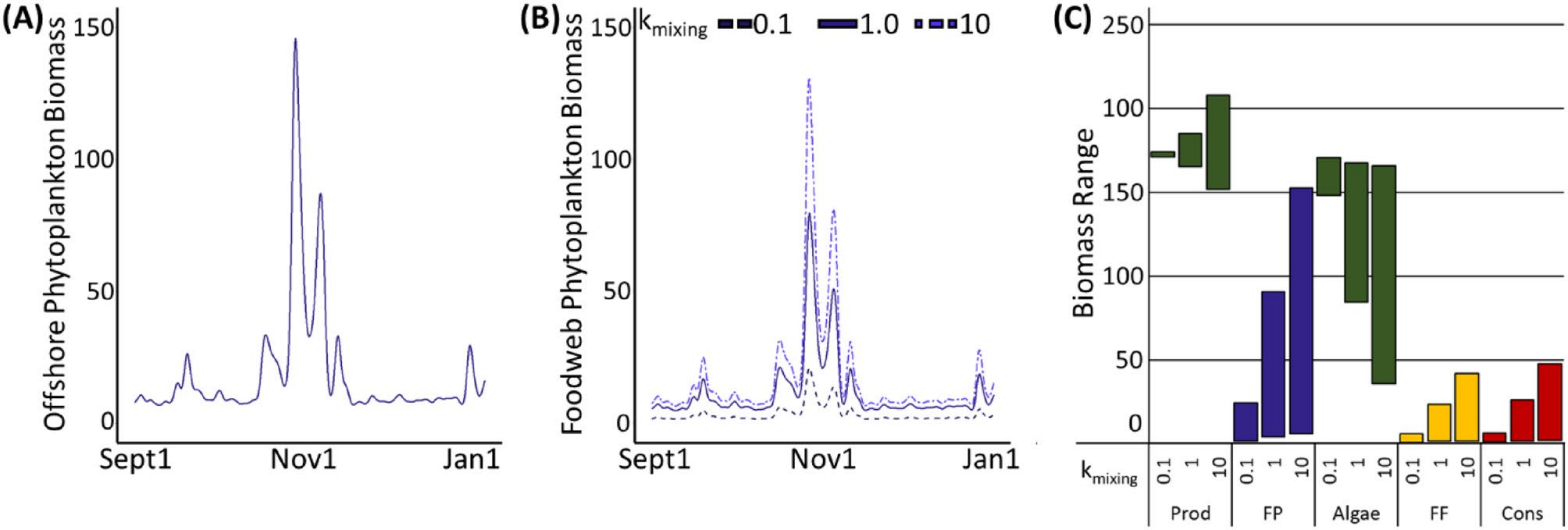
(**A**) Measured biomass of offshore phytoplankton from Sept 2003 to Jan 2004. (**B**) Simulated biomass of food web phytoplankton under k_mixing_ = 0.1 h^−1^ (dashed line), 1.0 h^−1^ (solid line), and 10 h^−1^ (dot-dashed line). (**C**) Range of simulated biomasses from Sept 2003 to Jan 2004 for four trophic categories and three values of k_mixing_. This range spans the minimum simulated biomass to the maximum simulated biomass. *Prod* summed biomass of 46 producer species, *FP* food web phytoplankton biomass, *Algae* summed biomass of 45 algal species, *FF* summed biomass of 15 filter feeder species, *Cons* summed biomass of 60 consumer species. All biomasses are reported in kg/m^2^.

We simulated the effects of varying the pelagic-intertidal mixing rate on the rocky intertidal ecosystem by assigning values of 0.1, 1.0, and 10 h^−1^ to k_mixing_ (Fig. 3A,B). Although increasing values of k_mixing_ increased the concentration of phytoplankton in the rocky intertidal (Supplementary Fig. S5), our simulations showed only a small relative increase in total primary productivity due to the community-level carrying capacity shared by producer species (Fig. 3C, Prod). However, total consumer biomass—and biomass of filter-feeding invertebrates in particular—increased with k_mixing_, highlighting the importance of phytoplankton in fueling the intertidal ecosystem across trophic levels (Fig. 3C, FF and Cons).

**Figure 4:**
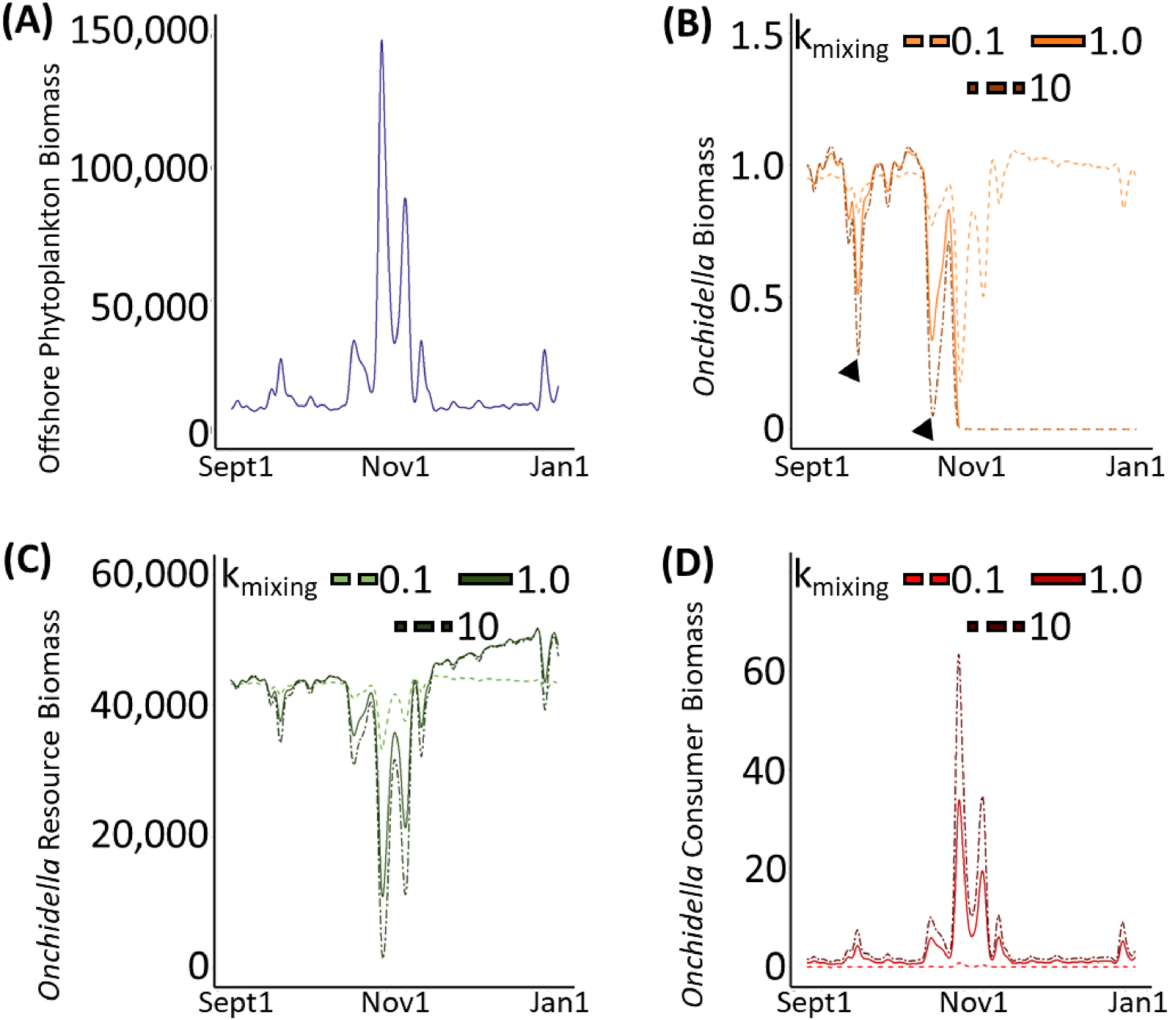
Results show simulations performed using empirical data from Sept 2003 to Jan 2004 under k_mixing_ = 0.1 h^−1^ (dashed lines), 1.0 h^−1^ (solid lines), and 10 h^−1^ (dot-dashed lines). (**A**) Measured biomass of offshore phytoplankton. (**B**) Simulated biomass of the sea snail *Onchidella*. (**C**) Sum of simulated biomasses for 14 algae species that *Onchidella* consumes. (**D**) Simulated biomass of *Acanthocyclus gayi*, *Onchidella*’s sole reported predator in Las Cruces. All biomasses are reported in g/m^2^.

The pelagic-intertidal mixing rate exhibits profound influence on the persistence of certain species, as highlighted by the response of the sea snail *Onchidella* to peaks in offshore phytoplankton (Fig. 4, Supplementary Fig. S2). Using an intermediate mixing rate (k_mixing_ = 1.0 h^−1^), our model predicts that the biomass of *Onchidella* would have reached a critical threshold in late October 2003 (Fig. 4B—solid line) caused by an increase in offshore phytoplankton (Fig. 4A) that produced a moderate reduction in *Onchidella’s* resources (algae, Fig. 4C—solid line) and a large increase in its predator’s biomass (Fig. 4D—solid line). The ATN model considers a species at risk of local extirpation when its calculated biomass drops below a critical threshold—in our case 10^–6^ g/m^2^^6,34^—because such extremely low biomass levels do not recover without an external input of biomass of such species. Local extirpation of *Onchidella* was simulated at k_mixing_ = 1.0 h^−1^ in six of the 12 years simulated (1999, 2000, 2002, 2003, 2006, and 2007). In all six of these years, offshore phytoplankton peaked at concentrations over 70,000 g/m^2^ (Table 1, Supplementary Fig. S2).

Decreasing the mixing rate to 0.1 h^−1^ results in slower exchange between intertidal habitat and offshore waters and rescues *Onchidella* from extirpation (Fig. 4B—dashed) because the biomasses of its resources (Fig. 4C—dashed) and predator (Fig. 4D—dashed) are significantly less affected by offshore phytoplankton. By lowering our simulated pelagic-intertidal mixing rate, *Onchidella* was rescued from local extirpation in all six of the years with high offshore phytoplankton (Table 1, Supplementary Fig. S2). Conversely, increasing the mixing rate to 10 h^−1^ results in almost immediate exchange with offshore waters and exacerbates the extirpation that was observed for intermediate mixing with both a stronger reduction in *Onchidella*’s resources (Fig. 4C—dot-dashed) and a more dramatic increase in its predator biomass (Fig. 4D—dot-dashed). The biomass of *Onchidella* itself was much more variable under higher mixing rates with two additional incidents when *Onchidella* was at critical risk of extirpation (Fig. 4B—dot-dashed, triangles). Simulations performed at this elevated pelagic-intertidal mixing rate predicted a local extirpation of Onchidella in four additional years which showed only intermediate levels of offshore phytoplankton between 47,500 and 70,000 g/m^2^ (2001, 2004, 2005, and 2008) (Table 1, Supplementary Fig. S2).

In our simulations, the survival of *Onchidella* was influenced by two factors: the peak abundance of offshore phytoplankton and the choice of pelagic-intertidal mixing rate. Our simulations indicate (1) that *Onchidella* can survive during periods with relatively modest peaks in offshore phytoplankton at all rates of pelagic-intertidal mixing, and (2) that *Onchidella* can survive scenarios with low pelagic-intertidal mixing rates regardless of the intensity of fluctuations in offshore phytoplankton abundance (Table 1, Supplementary Fig. S2). However, during years with intermediate peaks in offshore phytoplankton, *Onchidella* faced extirpation when subjected to high rates of pelagic-intertidal mixing (k_mixing_ = 10 h^−1^). Further, during years with very high peaks in offshore phytoplankton abundance, *Onchidella* experienced extirpation at both high (k_mixing_ = 10 h^−1^) and intermediate (k_mixing_ = 1.0 h^−1^) levels of pelagic-intertidal mixing. This can be explained by the modelled effects of elevated foodweb phytoplankton has on summed algal biomass. Since both increased k_mixing_ and elevated offshore phytoplankton act to increase foodweb phytoplankton concentrations, both factors will suppress the biomass of algal species. If the biomass of algal species drops too low, Onchidella is effectively resourceless and put at risk of extirpation (Fig. 3C). We observe that *Onchidella’s* survival is modulated in a dose-dependent manner by peak abundance of offshore phytoplankton and that an appropriate combination of peak offshore phytoplankton abundance and pelagic-intertidal mixing rate is necessary for its survival.

### Variation from phytoplankton subsidy is most pronounced in consumers and is exacerbated by higher pelagic-intertidal mixing rates

**Figure 5:**
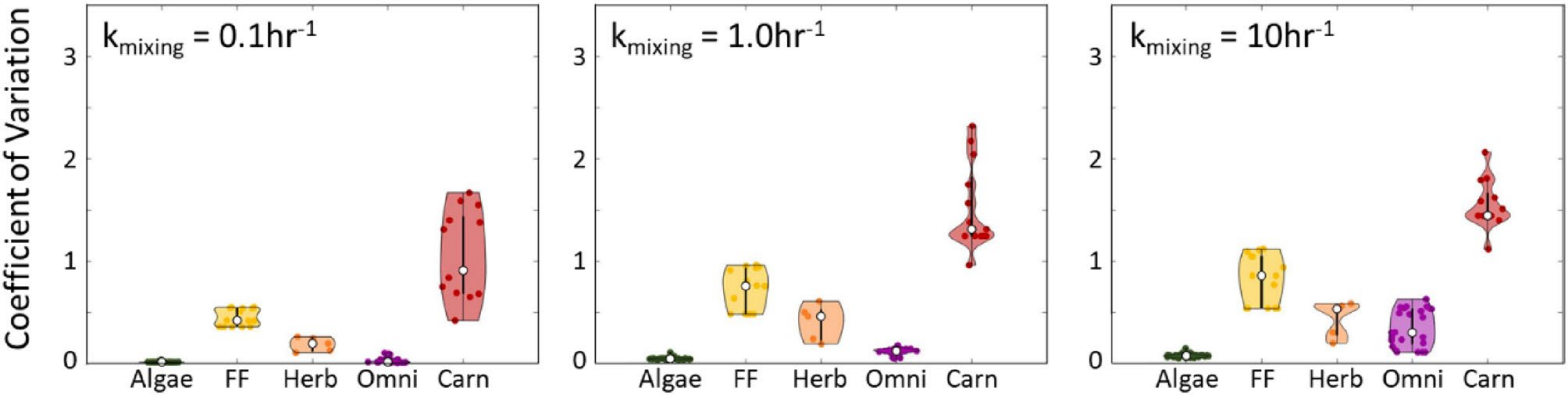
Violin plots showing the coefficients of variation for each species normalized to that of offshore phytoplankton. Results shown for 2003 and each value of k_mixing_ investigated. Species were grouped into one of five guilds: algae (Algae), filter feeders (FF), herbivores (Herb), omnivores (Omni), and carnivores (Carn).

Within-year variations in species biomasses caused by fluctuations in offshore phytoplankton concentration differed across species guilds. To assess the variability across the network, each species was assigned to one of five guilds: algae, filter feeders, herbivores, omnivores, and carnivores. Variability within each guild was evaluated using a species-specific coefficient of variation that was normalized against the variability in offshore phytoplankton (Fig. 5).

For each rate of pelagic-intertidal mixing, the algal guild exhibited the lowest normalized coefficient of variation. The high biomass of algae and kelp causes the variation-to-mean ratio to be small relative to that of phytoplankton. Herbivore species also exhibited relatively low normalized coefficients of variation, explained by their tight trophic relationship with non-planktonic producer species.

Filter feeders, omnivores, and carnivores exhibited differential patterns of variability under different scenarios of pelagic-intertidal mixing. As the pelagic-intertidal mixing rate parameter was raised, variability in the biomasses of filter feeders increased. This can be attributed to two factors: (1) the strong trophic connection between filter feeders and the abundance of phytoplankton in the food web and (2) our previous result showing that higher values of pelagic-intertidal mixing rate enhance the availability of phytoplankton as a food source for filter feeders. Together, these two factors increase variability in filter feeder abundances, pushing variability closer to that of offshore phytoplankton. Variability arising from fluctuations in offshore phytoplankton abundance is more ambiguous in omnivore species. Omnivores exhibit extremely low relative variability under low pelagic-intertidal mixing rates, with relative variability increasing dramatically with mixing rate. Variability in carnivore species’ biomasses, however, shows elevated relative variability even in scenarios with low pelagic-intertidal mixing rate. Further, carnivores show a clear trend of increased variability with increasing pelagic-intertidal mixing rate. Carnivores tend to have low total biomasses and incorporate several different sources of variability due to their position near the top of the food web, causing their biomass variability to be large. These observations are consistent with previous studies showing that predators amplify biomass fluctuations and exhibit high sensitivity to network perturbations^44^.

## Discussion

The rich and diverse nearshore ecosystems of the rocky shore would not be possible without the subsidy provided by offshore phytoplankton^3,45^. However, the rate at which pelagic phytoplankton is delivered to intertidal habitats is highly variable, depending on both the concentration of offshore phytoplankton, which can vary by more than two orders of magnitude in upwelling systems, and the variability in pelagic-intertidal mixing rates, which are driven by a combination of waves, tides, and wind-driven currents.

Traditional ATN modeling using a stationary approach or a constant rate of subsidy does not capture the population dynamics that result from variable phytoplankton input to the network nor the food web level consequences of the different pelagic-intertidal mixing rates. Several recent publications have sought to incorporate time-dependent abiotic forcing into trophic network modeling using either wholly or partially simulated parameters including carrying capacity, intrinsic growth rate of producers, and metabolic rate of consumers among others^46–48^. Our work advances population dynamic modeling by developing a novel ATN methodology using an empirical time-series of chlorophyll-a to realistically simulate time-dependent subsidy (allochthonous input) in nearshore foodweb structure. This methodology allowed us to discover different responses to the variability in phytoplankton delivery that depends on the position of each species in the food web—and to relate those responses to oceanographic events reflected in the empirical time-series of chlorophyll-a.

A principal finding is that incorporating pulsed, time-dependent subsidy of phytoplankton into our population dynamics models influences transient food web dynamics and leads to important temporal fluctuation in species’ biomasses. Moreover, our model suggests that the local rate of delivery of pelagic phytoplankton to the intertidal can amplify or alleviate the magnitude of these fluctuations, having important consequences on species’ risk of extinction as illustrated by our results with *Onchidella*. High delivery rates essentially increase the magnitude of the subsidy pulse and thus amplify the magnitude of transient effects leading to greater temporal variability in populations. Our results reinforce those of others^44^ that report carnivores, as a multispecies functional group, are particularly sensitive to perturbations, showing the overall greatest cumulative amplification of variation in biomass fluctuations due to elevated phytoplankton abundances. For intermediate species, this can lead to intensification and further amplification of top-down effects. For herbivore species not buffered by increased bottom-up effects, our simulations imply that the boost in phytoplankton pulse size can lead to sufficient intensification of top-down, negative effects that it pushes the population over a threshold of low biomass to a point that it cannot recover. These results are consistent with experimental observations showing increased risk of extirpation in meso-predator populations due to an imbalance in top-down and bottom-up forcing^49–51^. For these, variation in risk to extirpation depends on network connectivity and predator identity. In contrast, under similar pelagic phytoplankton abundance, simulations performed using lower mixing rates can buffer these populations from extinction by isolating them from offshore fluctuations and thus favor more balanced top-down/bottom-up effects that dampen biomass fluctuations.

Consistent with the work by Avila-Thieme et al.^6^ using the same network model but with constant subsidy rates, we found that increased phytoplankton biomass decreases the biomass of non-planktonic producers via competition for the shared carrying capacity among producers. Additionally, both models found that increased phytoplankton biomass increased the biomass of filter feeders and that of their predators but decreased that of herbivores via the combined effects of increased predation (more abundant predators shared with filter feeders) and decreased resource availability (non-planktonic producers). Finally, both models found that omnivore species demonstrate greater resilience to elevated phytoplankton levels than herbivores by exploiting additional resources that are not affected by the decline in non-planktonic producers. This consistency between the two models suggests that these food web responses to increased phytoplankton subsidies are robust to the transient dynamics caused by the time-dependent pulsing of subsidies. Rather, these transient dynamics predict species extirpations, especially at higher trophic levels, that the model does not show using constant subsidy rates. We note these extirpations occur despite model baseline phytoplankton levels that support all species in steady state. Increased risk of extinction under transient dynamics is particularly important for understanding the effects that extreme oceanic events driven by climate change may cause to intertidal ecosystems.

No model completely represents all dynamics in a real system, and there are several points where additional empirical data may improve our simulations. The ATN model as used here only accounts for network structure, bioenergetic and trophic relationships between species, and empirical time-series data on chlorophyll-a concentration. Our choices of fluorescence scaling and pelagic-intertidal mixing rate rely on the previous model’s subsidy value and may be improved using oceanographic simulations or empirical data. Without any available data recording the pelagic-intertidal mixing of nearshore phytoplankton, we estimate surf-zone flushing times from prior studies of circulation and dye concentrations^52,53^. We incorporate detritus through a baseline particulate organic matter concentration that supports filter feeders during extended periods of low offshore phytoplankton. While this dampens biomass fluctuations, they are sufficient to show significant results as demonstrated through our analysis of *Onchidella*. Additionally, there are several other biotic and abiotic factors that are not incorporated in our model—most importantly, sea surface temperatures, which modulate the biological rates underpinning ATN modeling, and nutrient concentration, which controls algal growth rates.

Further, to fully assess the vulnerability of food webs to time-varying subsidies, there is a need to account for the different time scales of specific trophic processes in relation to the time scales of environmental subsidies. For example, time-dependent changes in feeding and assimilation processes are not included in our model. Feeding and the capacity to capitalize on accessible prey can exhibit substantial variability owing to alternative saturation rates or shifts in behavior and foraging tactics aimed at striking a balance between risk and reward in the presence of multiple stressors. Additionally, differences in the timing of resource peaks may further contribute to the intricacies of this variability. Thus, future work to incorporate the relative timing of plankton subsidy to that of temporal variability of foraging could prove insightful to the sensitivity of propagating effects and whether timing-mismatches are enough to alter the relative balance of top-down and bottom-up effects.

Our study introduces a novel methodology incorporating empirical timeseries data into the ATN framework to study the effects of variable abundances of oceanic phytoplankton on species in a Chilean rocky intertidal ecosystem. Our results draw attention to the importance of transient population dynamics and the role they may play in predicting species extirpations during extreme events. Specifically, large fluctuations in offshore phytoplankton biomass put intertidal species at risk of local extirpation even though levels of intertidal phytoplankton were not allowed to drop below a level that supported the persistence of all species in steady-state simulations. Our results suggest that for a species to persist in the face of large-scale biomass variability introduced by bottom-up processes, that a concomitant top-down, network-scale process is required to dampen biomass oscillations allowing for species persistence.

## Methods

### Chlorophyll-a dataset and preparation

Nearshore chlorophyll-a density (${\delta }_{chloro}$ g/m^3^) was measured daily from a permanent sampling quadrat at the Estación Costera de Investigaciones Marinas (ECIM) in Las Cruces, Chile between Jan 1999 and Dec 2010^28,54^. We applied a linear approximation with slope ${\beta }_{1}$ and intercept ${\beta }_{0}$ to transform chlorophyll-a density to phytoplankton population density (${D}_{phyto}$ individuals/m^3^). We used a frequency-weighted average body size (${m}_{phyto}$ g/individual) of several phytoplankton species present at the Las Cruces sampling site^6^ to convert from phytoplankton density to volumetric phytoplankton density (${\delta }_{phyto}$ g/m^3^) according to

$$\delta_{phyto} = m_{phyto} D_{phyto} = m_{phyto} \left( {\beta_{1} \delta_{chloro} + \beta_{0} } \right).$$

We normalized the total amount of phytoplankton subsidies to the time-variable model over the twelve-year study period to the amount of subsidies that would be delivered to the Avila-Thieme 2021 model over the same time interval^6^. Avila-Thieme 2021 uses a daily phytoplankton subsidy of $7355\frac{g}{{m}^{2}d}$. The Avila-Thieme 2021 model delivers a total phytoplankton subsidy, ${s}_{total}$, to a quadrat with area $A\, {m}^{2}$ of

$$s_{total} = \left( {7355\frac{g}{{m^{2} d}}} \right)\left( {A\, m^{2} } \right)\left( {365\frac{d}{yr}} \right)\left( {12 yr} \right).$$

The time-variable model provides phytoplankton subsidy to the volume under a quadrat with volume $V\, {m}^{3}$ and average depth ${z}_{ave}$. Assuming the foodweb compartment is rapidly depleted of chlorophyll to baseline, we calculate a total phytoplankton subsidy to the time-variable model of

$$\begin{aligned} s_{total} & = \left( {\mathop \sum \limits_{daily 1999}^{2010} \delta_{offshore} - \delta_{foodweb} } \right)\left( V \right) \\ & = m_{phyto} \left( {\mathop \sum \limits_{daily 1999}^{2010} (\beta_{1} \delta_{chloro} + \beta_{0} ) - \left( {\beta_{0} } \right)} \right)\left( A \right)\left( {z_{ave} } \right) \\ & = \alpha A\mathop \sum \limits_{daily 1999}^{2010} \delta_{chloro} , \\ \end{aligned}$$

where $\alpha$ is a new scaling factor representing three distinct physical properties: the mass of individual phytoplankton, the scaling from chlorophyll density to phytoplankton density, and the average depth of the water column under the quadrat. $\alpha$ has units of m^1^ according to

$$\alpha = \left( {m_{phyto} \frac{{g_{phytoplankton} }}{individual}} \right)*\left( {\beta_{1} \frac{individual}{{g_{chlorophyll} }}} \right)*\left( {z_{ave} m} \right).$$

Setting the total phytoplankton subsidies equal between the two models, we obtain a scaling factor of $\alpha =1.567 {\text{m}}.$ We scaled our empirical chlorophyll density time-series (g/m^3^) by alpha (m) to obtain an area-based approximation of phytoplankton biomass (${B}_{phyto}$ g/m^2^). A continuous spline was fit to the phytoplankton biomass time-series and was numerically differentiated to obtain the rate law governing the biomass of offshore phytoplankton, *B*_*op*_ (see *Network Structure Changes* under *Non-Autonomous ATN Extension* below).

## Allometric trophic network

The Las Cruces food web contains 106 species with 1,362 trophic links (Fig. 1A, ref.^55^). This foodweb includes all species found to co-occur during community structure surveys carried out at multiple, wave-exposed rocky intertidal sites along a ~ 700 km stretch of the central Chilean coast^16,56,57^. All 106 species are known to coexist at the Las Cruces site^6,57^.

Our ATN modeling utilizes two sets of differential equations to describe the biomass of each producer (Eq. 1) and consumer (Eq. 2) species (recorded in units of grams biomass per square meter) according to the principles of mass balance as follows:

1 $$\frac{{dB_{i} }}{dt} = \overbrace {{r_{i} B_{i} G_{i} \left( {\varvec{B}} \right)}}^{Autotrophic \,growth} - \overbrace {{ \mathop \sum \limits_{j} \frac{{x_{j} y_{ji} B_{j} F_{ji} \left( {\varvec{B}} \right)}}{{e_{ji} }}}}^{Loss\, to \,predation }$$

2 $$\frac{{dB_{i} }}{dt} = \overbrace {{f_{a} x_{i} B_{i} \mathop \sum \limits_{j} y_{ij} F_{ij} \left( {\varvec{B}} \right)}}^{Gain \,from \,consumption} - \overbrace {{f_{m} x_{i} B_{i} }}^{Metabolic\, loss} - \overbrace {{\mathop \sum \limits_{j} \frac{{x_{j} y_{ji} B_{j} F_{ji} \left( {\varvec{B}} \right)}}{{e_{ji} }}}}^{Loss \,to \,predation}.$$

3 $$G_{i} \left( {\varvec{B}} \right) = 1 - \frac{{\mathop \sum \nolimits_{j \in producers} B_{j} }}{K}.$$

4 $$F_{ij} \left( {\varvec{B}} \right) = \frac{{\omega_{ij} B_{j}^{q} }}{{B0_{ij}^{q} + d_{i} B_{i} B0_{ij} + \mathop \sum \nolimits_{l \in resources} \omega_{il} B_{l}^{q} }}.$$

The biomass of producer species changes according to the balance of autotrophic growth with predation (Eq. 1). Autotrophic growth is controlled by the intrinsic growth rate, r_i_, and current biomass, B_i_, and is scaled by a logistic growth factor, G_i_ (Eq. 3). The logistic growth factor takes into consideration the shared, community-level, carrying capacity for producer species, K, to slow producer growth as the summed producer biomass approaches or exceeds carrying capacity. Biomass loss to predator j from prey i increases with the predator’s mass-specific metabolic rate, x_j_, and prey-specific attack rate, y_ji_, and decreases with the assimilation efficiency of prey j into predator i, e_ji_.

The biomass of consumer species changes according to the balance of consumer growth rate, explicitly modeled metabolic loss and mortality, and loss to predation. Consumer growth rate is modeled as assimilation of prey biomass; consequently, consumer growth rates are a function of their total consumption rate, ${x}_{i}{B}_{i}\sum_{j\epsilon prey}{y}_{ij}{F}_{ij}({\varvec{B}})$, scaled by an assimilation coefficient, f_a_. Metabolic loss including mortality is directly proportional to current biomass and metabolic rate and is scaled by a mortality coefficient, f_m_. Finally, the loss to predation term is identical to that of producers.

The Holling’s functional response, F_ij_, is used to determine the consumption rate of each resource for each consumer (Eq. 4). This function considers the relative preference of consumer i for resource j, ω_ij_, as well as the Holling’s coefficient, q, to determine curve shape. We invoke an intermediate Holling’s Type II and Type III response by using q = 1.2^58^.

Supplementary Table S3 shows all model parameters with their initial values and equilibrium values. The Las Cruces ATN model was parameterized as in ref. 6. Intrinsic growth rate of producers, metabolic rate of consumers and maximum consumption rate of consumers were parameterized using measures of each species’ body size^6,55^. Initial biomass values were estimated using species density (mobile species and cnidaria) and surface cover (sessile species) from six years of empirical records maintained at the research site^6^. Community-level carrying capacity was estimated as the sum of biomasses for the fastest growing species in each of six producer functional groups (microalgae, ephemeral, corticate, crustose, coralline, and kelp) weighted by the number of species in each group^6,59^. We investigated the effects of varying the community-level carrying capacity on community composition (Supplementary Fig. S6). Half-saturation density parameters were estimated from past literature using the Lake Constance ATN model as a primary reference^6,32,60,61^.

### Non-autonomous ATN extension

*Parameter Modifications:* We forced our ATN model to use a timestep equal to one hour. Consequently, we converted intrinsic growth rates of all producers, r_i_, metabolic rates of all consumers, x_i_, and maximum consumption rates of consumers, y_i_, to an hourly rate using the daily rates reported in ref.^6^. All other parameters, including initial conditions, are identical to those used in ref.^6^.

*Network Structure Modifications:* To encapsulate the phytoplankton dynamics of this system, we split the existing phytoplankton node into three nodes: offshore phytoplankton, foodweb phytoplankton, and “baseline phytoplankton” representing detritus.

1. The biomass for offshore phytoplankton, ${B}_{op}$, is equal to the scaled, empirical dataset described in *Chlorophyll-a Dataset and Preparation*. This node connects to the food web only by water-borne exchange with the foodweb phytoplankton node. This introduces a new rate constant, k_mixing_, which controls the rate at which plankton moves from offshore into foodweb phytoplankton. k_mixing_ is assigned values of 0.1, 1.0, and 10 h^−1^ as denoted.
2. We separated the existing phytoplankton node into two nodes, baseline phytoplankton and food web phytoplankton. These two nodes share identical connections within the food web.

The biomass of “baseline phytoplankton” (representing detritus contribution to particulate organic matter available to filter feeders) is held constant at a value of 3,750 g/m^2^. This node supplies the minimal amount of phytoplankton required to prevent network collapse under steady state conditions (Supplementary Figs. S7, S8). This node represents organic particulate matter available for consumption by filter feeders such as detritus that is otherwise not modeled in our ATN.

The foodweb phytoplankton node provides the primary supply of phytoplankton to consumers in the food web (Supplementary Fig. S5). This node encapsulates the local dynamics of intertidal phytoplankton. Further, this node is subsidized by biomass from offshore phytoplankton via water-borne exchange (Eq. 5). This subsidy modifies the ATN foodweb phytoplankton according to the following relation.

5 $$\frac{{dB_{fp} }}{dt} = \left\{ {\begin{array}{*{20}l} {r_{fp} B_{fp} G_{fp} \left( {\varvec{B}} \right) - \mathop \sum \limits_{j} \frac{{x_{j} y_{j,fp} B_{j} F_{j,fp} \left( {\varvec{B}} \right)}}{{e_{j,fp} }} + k_{mixing} \left( {B_{op} - B_{fp} } \right), } \hfill & {B_{op} \ge B_{fp} } \hfill \\ {r_{fp} B_{fp} G_{fp} \left( {\varvec{B}} \right) - \mathop \sum \limits_{j} \frac{{x_{j} y_{j,fp} B_{j} F_{j,fp} \left( {\varvec{B}} \right)}}{{e_{j,fp} }}, } \hfill & { B_{op} < B_{fp} .} \hfill \\ \end{array} } \right.$$

### Simulations

Each year of empirical data, from Jan 1 to Dec 31, was analyzed independently. To better compare simulations performed under different empirical datasets, we sought to reduce the transient dynamics introduced by having initial conditions that were significantly different from runtime conditions. We evaluated two separate strategies for reducing the impacts of initial conditions and found little change between the two. Strategy 1 involved running simulations under an “equilibration phase” with the offshore phytoplankton node deactivated for ten years of simulation time prior to activating the empirical data during the “experimental phase” (Supplementary Fig. S9). Strategy 2 replaces the “equilibration phase” with ten appended copies of the empirical dataset. This allows population biomasses to approach runtime conditions more gently than strategy 1. Population distributions were consistent between each strategy, and the results presented here were obtained using strategy 1.

### Coefficient of variation and violin plots

We use the coefficient of variation, the ratio of standard deviation to mean, as the preferred metric of variability for the timeseries data presented here. We calculated coefficient of variation for each species during the time interval when its biomass is above the extinction threshold of 10^−6^ g/m^2^ (i.e., trailing zeroes were truncated where applicable), and normalized these values to the coefficient of variation that was calculated for the biomass of offshore phytoplankton.

Violin plots were used to display distributional data on coefficients of variation. These plots are a modification of box plots in that they show the median value, each individual value, interquartile range, and shade the kernel density estimate to give an indication of the data distribution. Wider sections of violin plots represent a higher probability that members in the population will take on a given value. Violin plots were generated in Matlab using code from ref.^62^.

### Supplementary Information

Supplementary Information.

## Data Availability

Simulation code and the Chilean intertidal data will be available upon acceptance at the repository https://github.com/Valdovinos-Lab/Chilean_Variable_Subsidy. The Chilean intertidal food web and species body sizes can also be found in^12^.

## References

[1] Krenz C (2011). Ecological subsidies to rocky intertidal communities: Linear or non-linear changes along a consistent geographic upwelling transition?. J. Exp. Mar. Biol. Ecol..

[2] Leslie HM (2005). Barnacle reproductive hotspots linked to nearshore ocean conditions. Proc. Natl. Acad. Sci..

[3] Menge BA (2003). Coastal oceanography sets the pace of rocky intertidal community dynamics. Proc. Natl. Acad. Sci..

[4] Phillips NE (2005). Growth of filter-feeding benthic invertebrates from a region with variable upwelling intensity. Mar. Ecol. Prog. Ser..

[5] Phillips NE (2007). A spatial gradient in the potential reproductive output of the sea mussel *Mytilus californianus*. Mar. Biol..

[6] Ávila-Thieme MI (2021). Alteration of coastal productivity and artisanal fisheries interact to affect a marine food web. Sci. Rep..

[7] Bracken ME (2012). Mussel selectivity for high-quality food drives carbon inputs into open-coast intertidal ecosystems. Mar. Ecol. Prog. Ser..

[8] Morgan SG, Shanks AL, MacMahan JH, Reniers AJ, Feddersen F (2018). Planktonic subsidies to surf-zone and intertidal communities. Ann. Rev. Mar. Sci..

[9] Largier JL (2006). WEST: A northern California study of the role of wind-driven transport in the productivity of coastal plankton communities. Deep Sea Res. Part II Top. Stud. Oceanogr..

[10] Largier JL (2020). Upwelling bays: How coastal upwelling controls circulation, habitat, and productivity in bays. Ann. Rev. Mar. Sci..

[11] Gomez FA (2017). Intraseasonal patterns in coastal plankton biomass off central Chile derived from satellite observations and a biochemical model. J. Mar. Syst..

[12] Largier, J. L. Rip currents and the influence of morphology on wave-driven cross-shore circulation. *Ref. Module Earth Syst. Environ. Sci.* 100–121 (2022).

[13] Morgan SG (2016). Surfzone hydrodynamics as a key determinant of spatial variation in rocky intertidal communities. Proc. R. Soc. B Biol. Sci..

[14] Shanks AL, Morgan SG, MacMahan J, Reniers AJ (2010). Surf zone physical and morphological regime as determinants of temporal and spatial variation in larval recruitment. J. Exp. Mar. Biol. Ecol..

[15] Hastings A (2018). Transient phenomena in ecology. Science.

[16] Blanchette CA, Wieters EA, Broitman BR, Kinlan BP, Schiel DR (2009). Trophic structure and diversity in rocky intertidal upwelling ecosystems: A comparison of community patterns across California, Chile, South Africa and New Zealand. Prog. Oceanogr..

[17] Gutiérrez D, Akester M, Naranjo L (2016). Productivity and sustainable management of the Humboldt Current large marine ecosystem under climate change. Environ. Dev..

[18] Montecino V, Lange CB (2009). The Humboldt Current system: Ecosystem components and processes, fisheries, and sediment studies. Prog. Oceanogr..

[19] Ochoa N, Taylor MH, Purca S, Ramos E (2010). Intra- and interannual variability of nearshore phytoplankton biovolume and community changes in the northern Humboldt Current system. J. Plankton Res..

[20] Chavez FP, Messié M (2009). A comparison of eastern boundary upwelling ecosystems. Prog. Oceanogr..

[21] Daneri G (2000). Primary production and community respiration in the Humboldt Current system off Chile and associated oceanic areas. Mar. Ecol. Prog. Ser..

[22] Ryther JH (1969). Photosynthesis and fish production in the sea: The production of organic matter and its conversion to higher forms of life vary throughout the world ocean. Science.

[23] Oyarzún D, Brierley CM (2019). The future of coastal upwelling in the Humboldt Current from model projections. Clim. Dyn..

[24] Martinez E, Antoine D, D’Ortenzio F, Gentili B (2009). Climate-driven basin-scale decadal oscillations of oceanic phytoplankton. Science.

[25] Thatje S, Heilmayer O, Laudien J (2008). Climate variability and El Niño southern Oscillation: Implications for natural coastal resources and management. Helgol. Mar. Res..

[26] Bakun A (1990). Global climate change and intensification of coastal ocean upwelling. Science.

[27] Sydeman WJ (2014). Climate change and wind intensification in coastal upwelling ecosystems. Science.

[28] Weidberg N (2020). Spatial shifts in productivity of the coastal ocean over the past two decades induced by migration of the Pacific Anticyclone and Ba’un’s effect in the Humboldt Upwelling Ecosystem. Glob. Planet. Change.

[29] Yodzis P, Innes S (1992). Body size and consumer-resource dynamics. Am. Nat..

[30] Brose U (2006). Consumer–resource body-size relationships in natural food webs. Ecology.

[31] Berlow EL (2009). Simple prediction of interaction strengths in complex food webs. Proc. National Acad. Sci..

[32] Boit A, Martinez ND, Williams RJ, Gaedke U (2012). Mechanistic theory and modelling of complex food-web dynamics in Lake Constance. Ecol. Lett..

[33] Brose U, Williams RJ, Martinez ND (2006). Allometric scaling enhances stability in complex food webs. Ecol. Lett..

[34] Schneider FD, Brose U, Rall BC, Guill C (2016). Animal diversity and ecosystem functioning in dynamic food webs. Nat. Commun..

[35] Albert G, Gauzens B, Loreau M, Wang S, Brose U (2022). The hidden role of multi-trophic interactions in driving diversity–productivity relationships. Ecol. Lett..

[36] Gauzens B, Rall BC, Mendonça V, Vinagre C, Brose U (2020). Biodiversity of intertidal food webs in response to warming across latitudes. Nat. Clim. Change.

[37] Glaum P, Cocco V, Valdovinos FS (2020). Integrating economic dynamics into ecological networks: The case of fishery sustainability. Sci. Adv..

[38] Perälä T, Kuparinen A (2020). Eco-evolutionary dynamics driven by fishing: From single species models to dynamic evolution within complex food webs. Evol. Appl..

[39] Uusi-Heikkilä S, Perälä T, Kuparinen A (2022). Fishing triggers trophic cascade in terms of variation, not abundance, in an allometric trophic network model. Can. J. Fish. Aquat. Sci..

[40] Brose U (2019). Predator traits determine food-web architecture across ecosystems. Nat. Ecol. Evol..

[41] Petchey OL, Beckerman AP, Riede JO, Warren PH (2008). Size, foraging, and food web structure. Proc. National Acad. Sci..

[42] Peters RH (1983). Size structure of the plankton community along the trophic gradient of Lake Memphremagog. Can. J. Fish. Aquat. Sci..

[43] Shurin JB, Gruner DS, Hillebrand H (2006). All wet or dried up? Real differences between aquatic and terrestrial food webs. Proc. R. Soc. B Biol. Sci..

[44] Berg S, Pimenov A, Palmer C, Emmerson M, Jonsson T (2015). Ecological communities are vulnerable to realistic extinction sequences. Oikos.

[45] Menge BA (2000). Top-Down and bottom-up community regulation in marine rocky intertidal habitats. J. Exp. Mar. Biol. Ecol..

[46] Kuparinen A, Perälä T, Martinez N, Valdovinos F (2019). Environmentally-induced noise dampens and reddens with increasing trophic level in a complex food web. Oikos.

[47] Eloranta AP, Perälä T, Kuparinen A (2023). Effects of temporal abiotic drivers on the dynamics of an allometric trophic network model. Ecol. Evol..

[48] Sauve AM, Barraquand F (2020). From winter to summer and back: Lessons from the parameterization of a seasonal food web model for the Białowieża forest. J. Anim. Ecol..

[49] Cavole LM (2016). Biological impacts of the 2013–2015 warm-water anomaly in the Northeast Pacific: Winners, losers, and the future. Oceanography.

[50] Jochum M, Schneider FD, Crowe TP, Brose U, O'Gorman EJ (2012). Climate-induced changes in bottom-up and top-down processes independently alter a marine ecosystem. Philos. Trans. R. Soc. B Biol. Sci..

[51] Burgos T, Salesa J, Fedriani JM, Escribano-Ávila G, Jiménez J, Krofel M, Virgós E (2023). Top-down and bottom-up effects modulate species co-existence in a context of top predator restoration. Sci. Rep..

[52] Smith JA, Largier JL (1995). Observations of nearshore circulation: Rip currents. J. Geophys. Res. Oceans.

[53] Clark LB, Ackerman D, Largier J (2007). Dye dispersion in the surf zone: Measurements and simple models. Cont. Shelf Res..

[54] Wieters EA (2003). Alongshore and temporal variability in chlorophyll a concentration in Chilean nearshore waters. Mar. Ecol. Prog. Ser..

[55] Kéfi S (2015). Network structure beyond food webs: Mapping non-trophic and trophic interactions on Chilean rocky shores. Ecology.

[56] Broitman BR, Navarrete SA, Smith F, Gaines SD (2001). Geographic variation of southeastern Pacific intertidal communities. Mar. Ecol. Prog. Ser..

[57] Wieters EA, Broitman BR, Brancha GM (2009). Benthic community structure and spatiotemporal thermal regimes in two upwelling ecosystems: Comparisons between South Africa and Chile. Limnol. Oceanogr..

[58] Williams RJ (2008). Effects of network and dynamical model structure on species persistence in large model food webs. Theor. Ecol..

[59] Lurgi M (2020). Grographical variation of multiplex ecological networks in marine intertidal communities. Ecology.

[60] Mulder C, Hendriks AJ (2014). Half-saturation constants in functional responses. Glob. Ecol. Conserv..

[61] Calbet A, Saiz E (2013). Effects of trophic cascades in dilution grazing experiments: From artificial saturated feeding responses to positive slopes. J. Plankton Res..

[62] Bechtold, B., Violinplot-Matlab. (2022). github, https://github.com/bastibe/Violinplot-Matlab

