# Supplementary material for: Modeling time-varying phytoplankton subsidy reveals at-risk species in a Chilean intertidal ecosystem
Source: Sci Rep. 2024 Mar 24;14:6995. doi: 10.1038/s41598-024-57108-9 (PMC10961311; doi:10.1038/s41598-024-57108-9)

**Modeling time-varying phytoplankton subsidy reveals at-risk species in a Chilean intertidal ecosystem**

Casey S. Duckwall ^a,b^, John L. Largier ^a,c^, Evie A. Wieters ^d^, Fernanda S. Valdovinos ^a^

**Supplemental Materials**

**Supplementary Table S1** – Table describing empirical dataset and simulation results performed under k_mixing_ = 1.0hr^-1^ (intermediate pelagic-intertidal mixing rate). Table shows year, number of days with data collection, percentage of year with data coverage, largest gap in data collection measured in days, the number of species predicted to have gone locally extinct, and their network ID number. Supplementary Table S2 details these species.

**
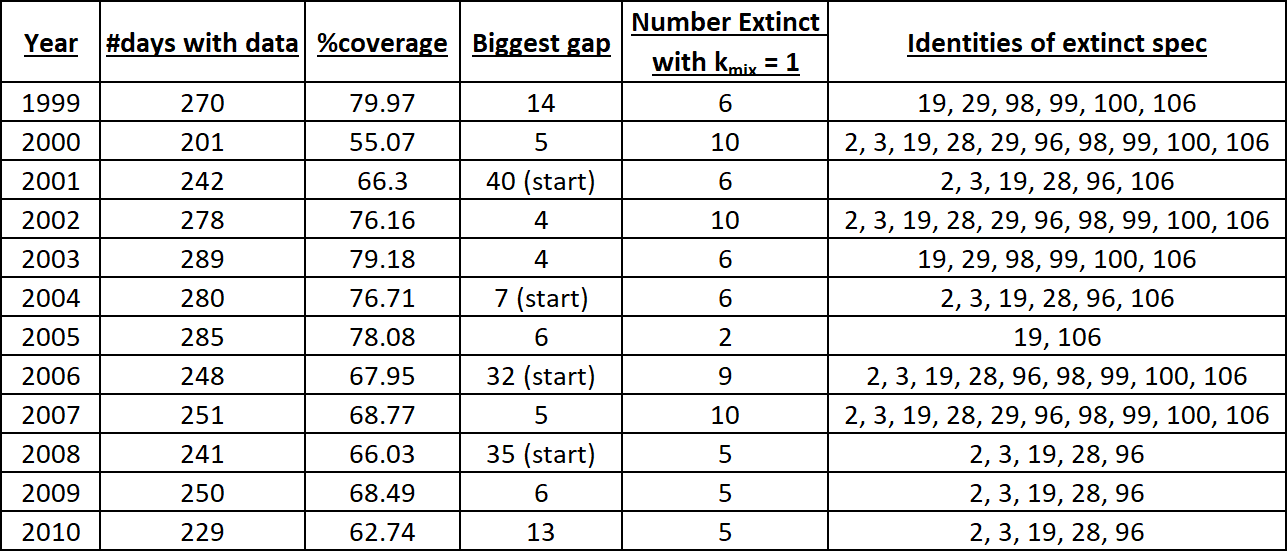
**

**Supplementary Table S2** – Table describing species that were predicted to have gone extinct in simulations performed under k_mixing_ = 1.0hr^-1^. For each species, the table lists network ID, scientific name, colloquial name, trophic guild, and number of years in which it was simulated to have gone extinct.

**
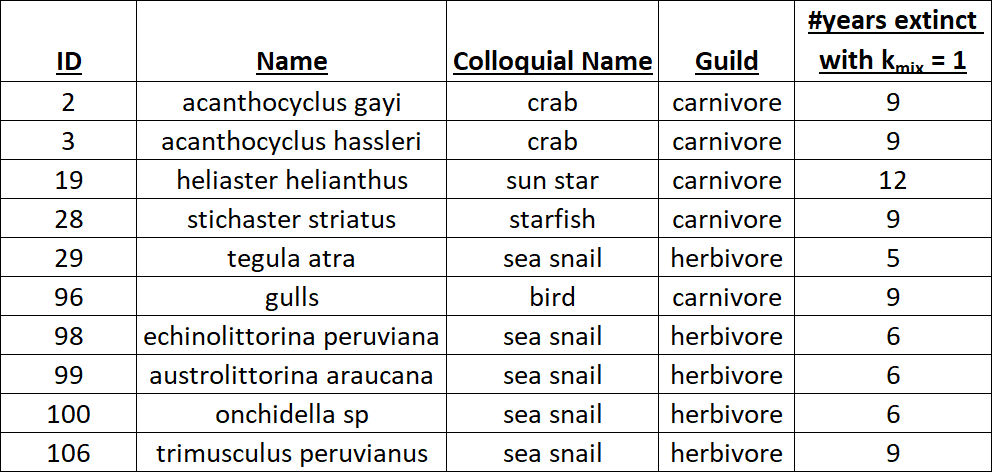
**

**Supplementary Table S3 -** Initial parameter values (IV) and equilibrium parameter values (EV) (see Fig S9) used in our version of the Allometric Trophic Network (ATN) model of the central Chilean coast. Initial parameter values are taken from Ávila-Thieme 2021, and equilibrium values are reported following equilibration after 10 years of simulation time.

| Parameter or variable | Units | Definition | Initial and Equilibrium Values min, max |
| --- | --- | --- | --- |
| B | g / m^2^ | Population density | IV: 1.24 x 10^-4^, 112,107  EV: 3.26 x 10^-4^, 4,401.6 |
| r | hr^-1^ | Mass-specific growth rate  of producers | 0.1075, 3.76 |
| x | hr^-1^ | Mass-specific growth rate  of consumers | 0.7284, 70.96 |
| G | - | Logistic growth factor (function) | IV: 0, 0.2495  EV: 8.99 x 10^-4^, 0.0259 |
| y | - | Attack rate | 1, 5.8 |
| F | - | Functional response  (function) | IV:1.23 x 10^-11^, 0.7154  EV: 1.17 x 10^-8^, 0.3622 |
| f_a_ | - | Fraction of biomass  assimilated by consumers | 0.4 |
| f_m_ | - | Fraction of biomass  lost to metabolic maintenance | 0.1 |
| e | - | Assimilation efficiency | 0.45, 0.85 |
| K | g / m^2^ | Community-level carrying capacity for producers | 176,299 |
| k_mixing_ | hr^-1^ | Pelagic-intertidal mixing rate | 0.1, 10 |

**Supplementary Figure S1** – Results shown mirror those in Figure 2. Each year has four associated panels – “Phytoplankton Abundance” shows biomass of both offshore phytoplankton (OP) and Food web Phytoplankton (FP); “Barnacle Abundance” shows biomass of the barnacle *B. laevis* (BL); “Producer Abundance” shows the summed biomass of all producer species (Prod) and the summed biomass of all algal species (Algae); and “Sea Snail Abundance” shows biomass of the omnivore sea snail *L. orbignyi* (LO) and the herbivore sea snail *T. atra* (TA). All panels show results using an intermediate pelagic-intertidal mixing rate parameter, k_mixing_ = 1.0hr^-1^.


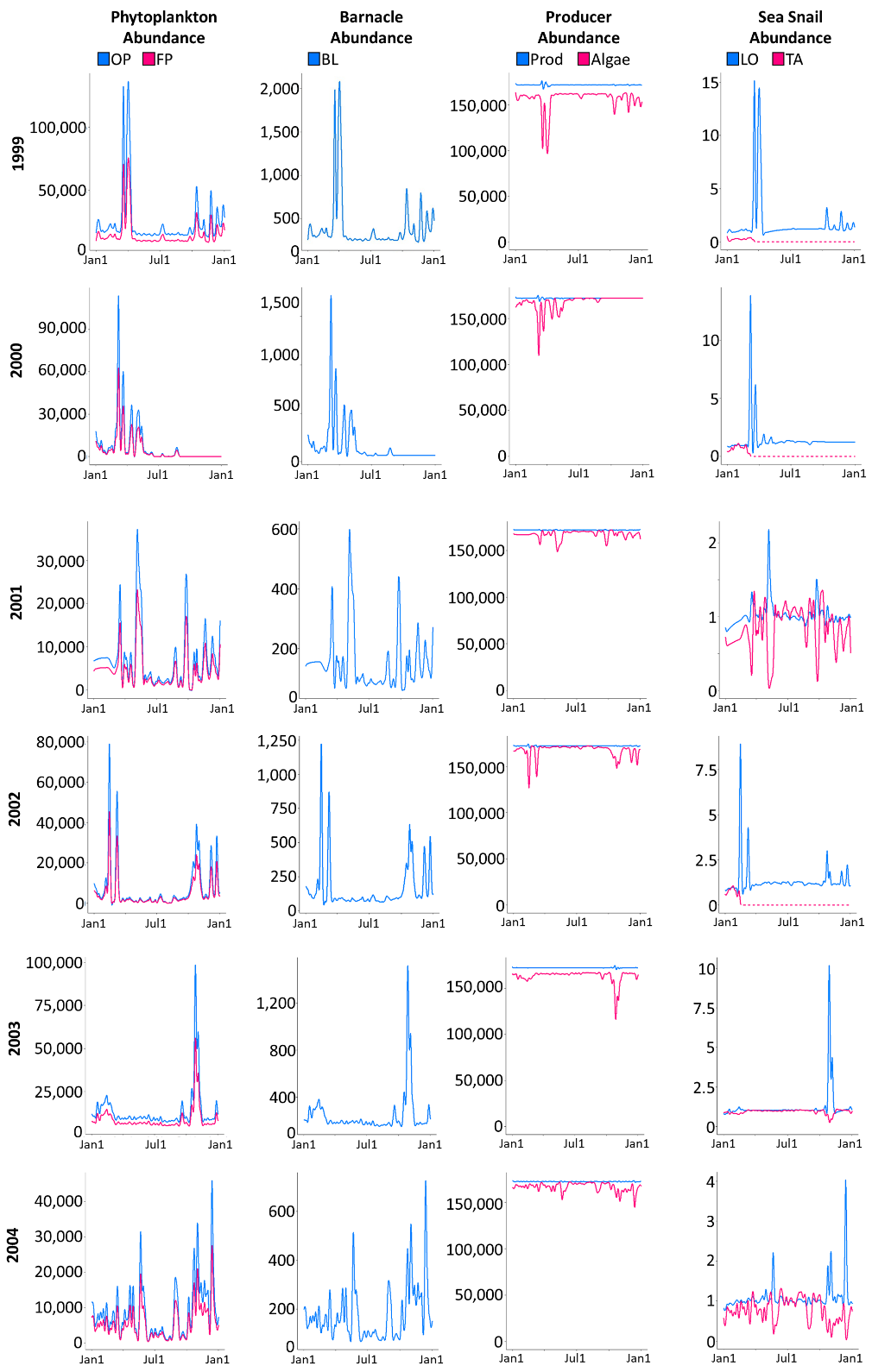


**
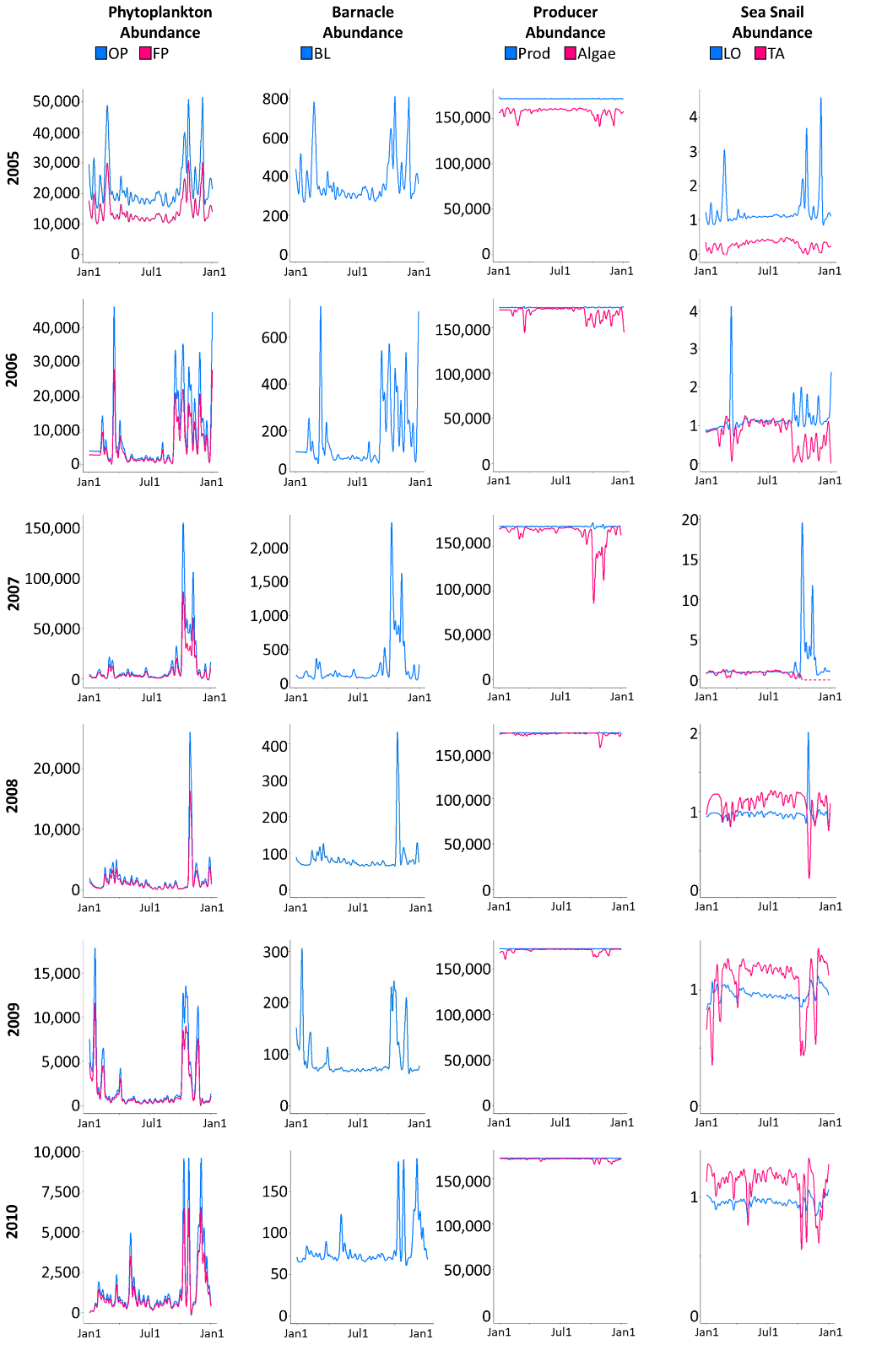
**

**Supplementary Figure S2** – Results shown mirror those in Fig. 4AB and are summarized in Table 1. Each year has two associated figures – top panel: offshore phytoplankton biomass, bottom panel: simulated *Onchidella* biomass under k_mixing_ = 0.1hr^-1^ (teal), 1.0hr^-1^ (green), and 10hr^-1^ (navy). Time-axes were truncated to show 3 month periods, with at least 1 month on either side of extirpation events. 2009 and 2010 show results from the full year because neither year’s simulation showed extirpations.


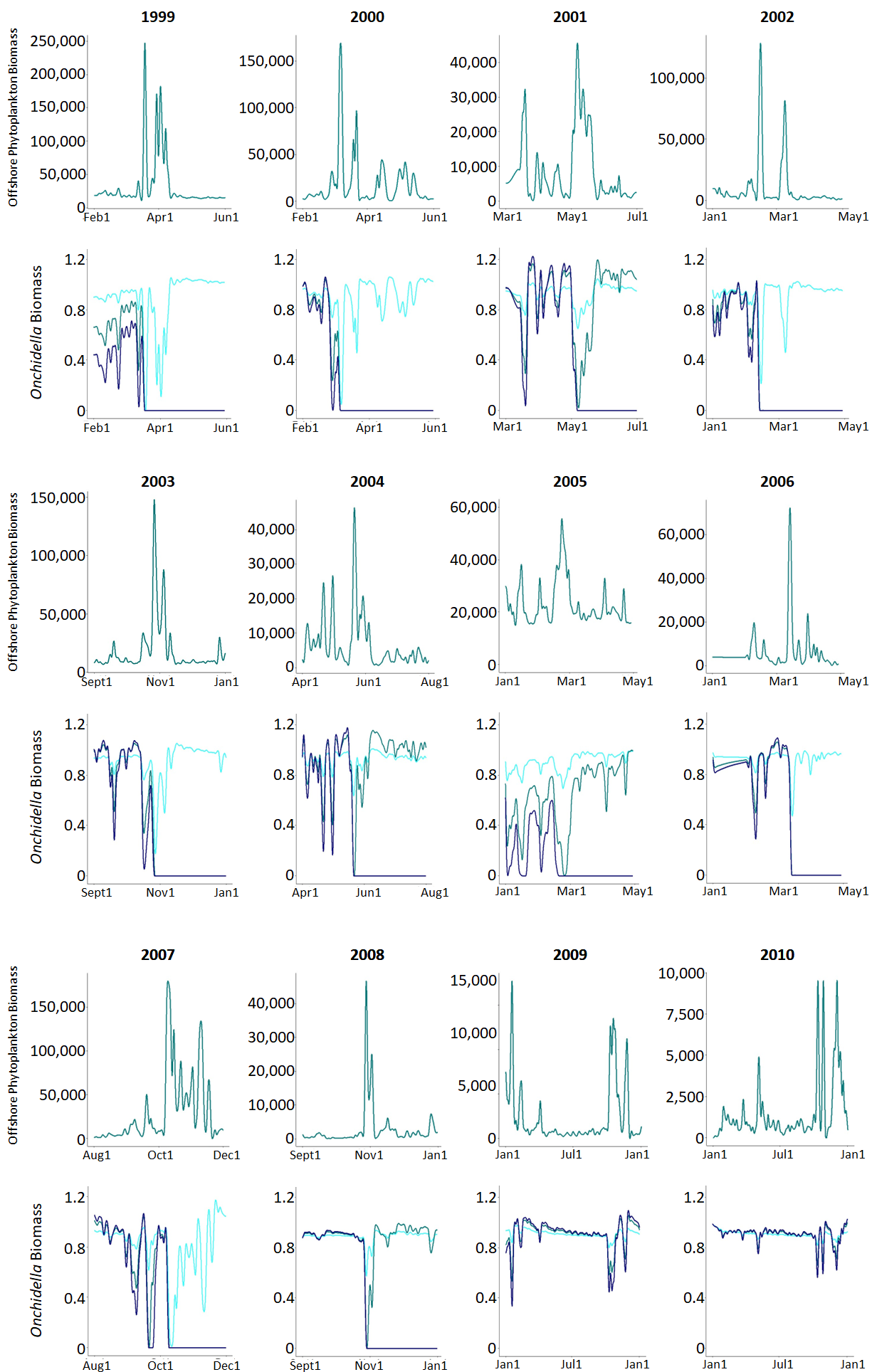


**Supplementary Figure S3 –** Relative abundance of all 15 filter feeders as they responded to offshore phytoplankton in 2003 presented in Figure 2. Each species’ biomass was normalized to its baseline level. These curves include 5 barnacles, 3 mussels, 1 worm, 1 tunicate, and 5 crabs. All biomass curves show positive responses to elevated offshore phytoplankton.

**
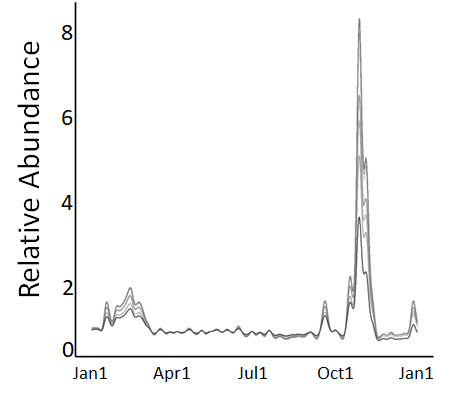
**

**Supplementary Figure S4 –** Relative abundance of all 20 sea snails as they responded to offshore phytoplankton in 2003 presented in figure 2. Each species’ biomass was normalized to its baseline level. These curves include 16 omnivorous sea snails (blue curves) and 4 herbivorous sea snails (green curves). All omnivorous sea snails show positive responses to elevated offshore phytoplankton, while all herbivorous sea snails show negative responses to elevated offshore phytoplankton.

**
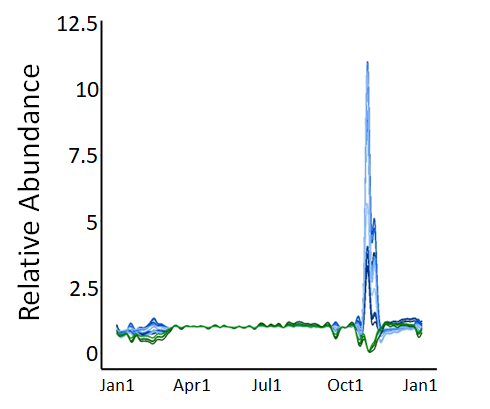
**

**Supplementary Figure S5** – Relative contribution to the total available intertidal phytoplankton (foodweb phytoplankton + baseline phytoplankton) as derived from baseline vs local dynamics vs subsidy simulated using the 2003 empirical data for offshore phytoplankton. As k_mixing_ increases, the fraction of subsidy-derived phytoplankton increases. As the subsidy-derived phytoplankton is incorporated into the intertidal phytoplankton pool, the fraction of phytoplankton arising from local dynamics also increases.


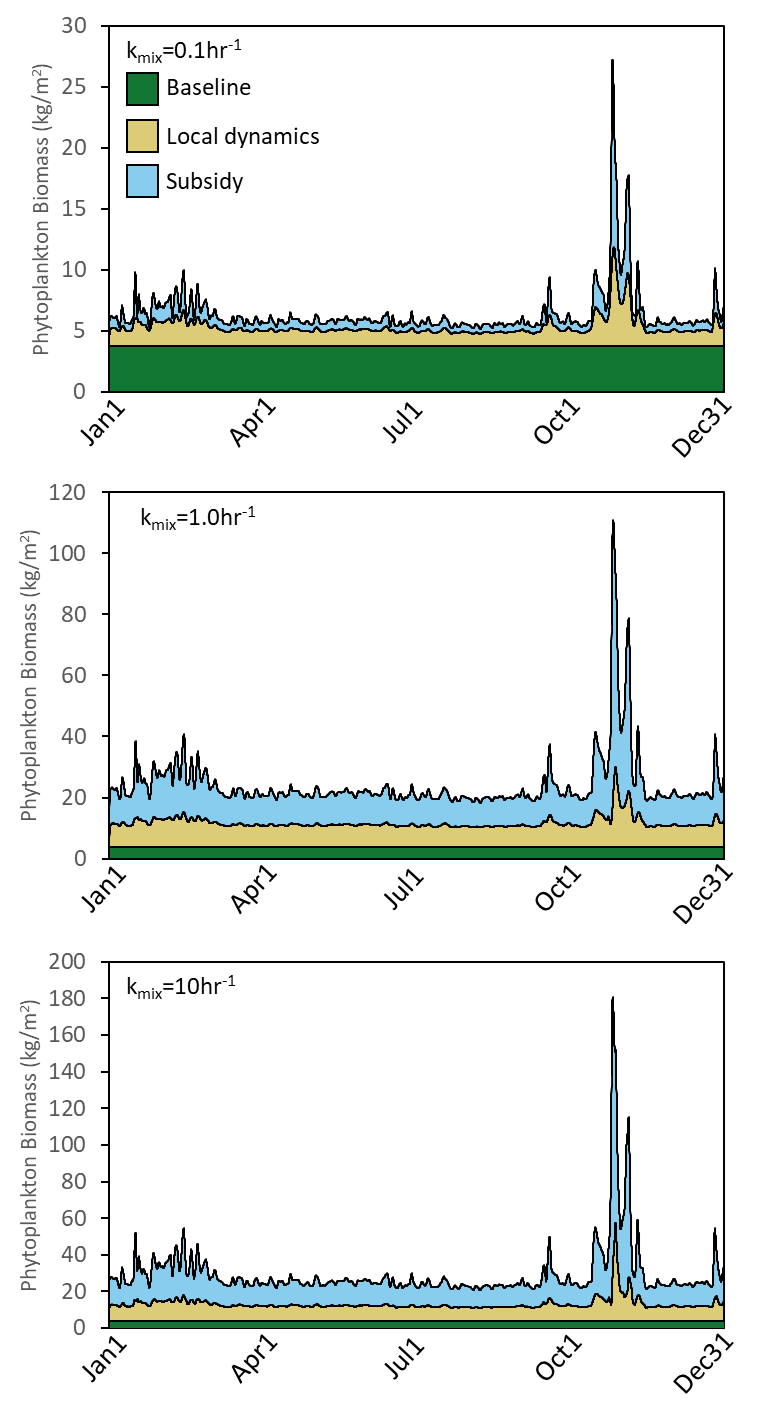


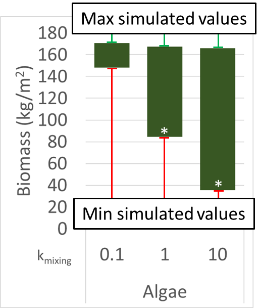
**Supplementary Figure S6** –Range of simulated biomasses (min to max) from Sept 2003 to Jan 2004 for four trophic categories – food web phytoplankton (FP), algae (Algae), filter feeders (FF) consumers (Cons) – three values of kmixing – 0.1, 1.0, 10.0 hr^-1^ – and seven values of carrying capacity – 10%, 25%, 50%, 100%, 200%, 400%, 1000%. Asterisks indicate simulations in which Onchidella was predicted to reach extinction.


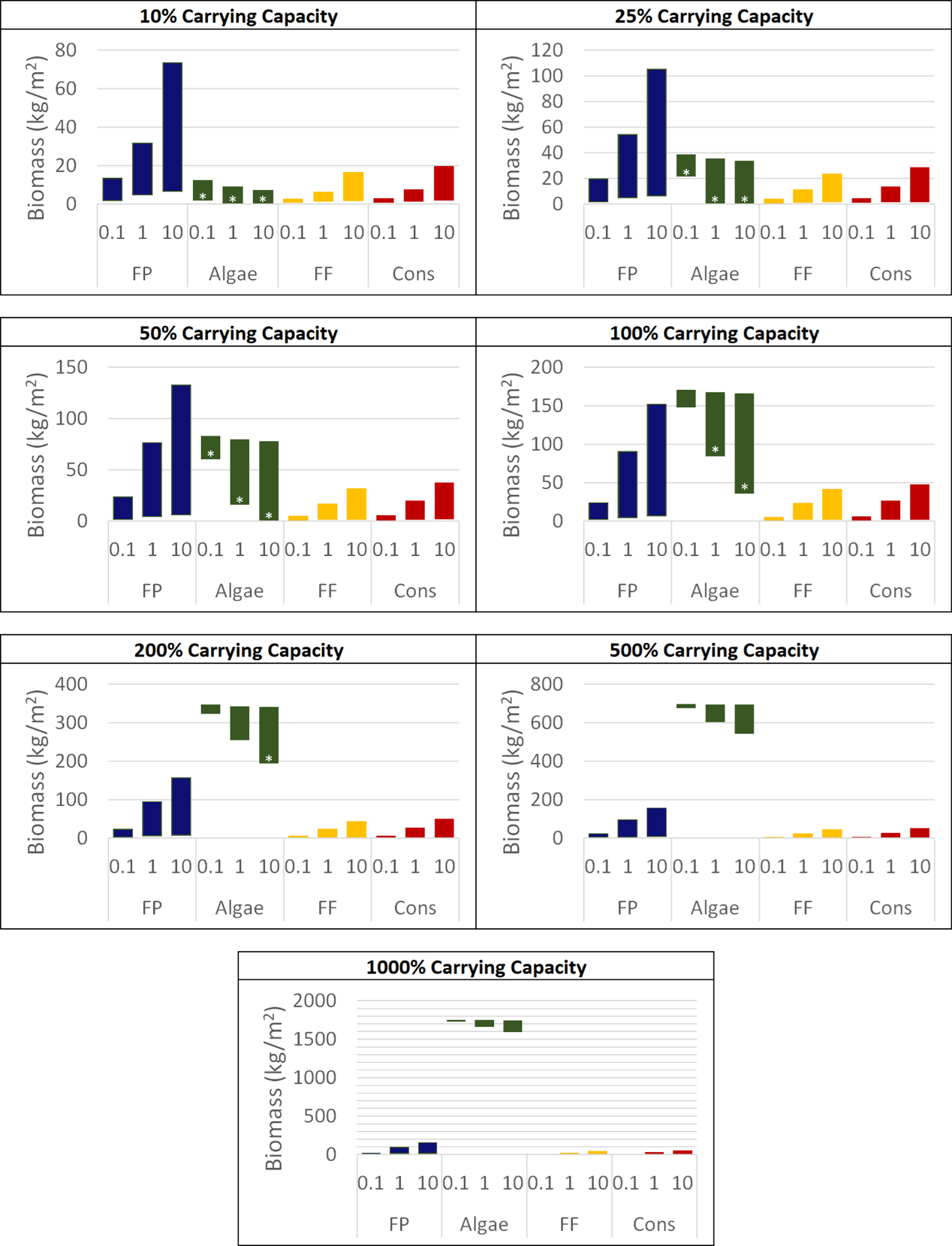


**Supplementary Figure S7** – A sweep of baseline phytoplankton values was conducted in the absence of offshore phytoplankton by varying baseline phytoplankton in increments of 250 g/m^2^. Baseline phytoplankton values of 3,750 g/m^2^ and higher were required to maintain all 106 species at the Las Cruces site. Baseline phytoplankton values of 3,500g/m^2^ and lower saw increasing numbers of species extinctions. The species extinctions were as follows:

3,500: Heliaster helianthus, Gulls

3,250: All prior plus Acanthocyclus gayi

3,000: All prior

2,750: All prior plus Acanthocyclus hassleri

2,500: All prior plus Stichaster striatus

2,250: All prior plus Concholepas concholepas

2,000: All prior

1,750: All prior plus Cinclodes nigrofumosus

1,500: All prior

1,250: All prior plus Acanthina monodon

1,000: All prior plus Anthotoe spp., Bunodactis spp., Oulactis concinnata,

Parantheopsis spp., Phymactis spp.

750: All prior

500: All prior plus Allopetrolisthes punctatus, Petrolisthes spinifrons, Petrolisthes angulosus,

Petrolisthes tuberculatus, Petrolisthes tuberculosus.

250: All prior

0: All prior plus Austromegabalanus Psittacus, Balanus laevis, Jhelius cirratus,

Notobalanus flosculus, Notochthamalus scabrosus, Brachidontes granulate,

Perumytilus purpuratus, Semimytilus algosus, Phragmatopoma spp., Pyura chilensis, Trimusculus peruvianus


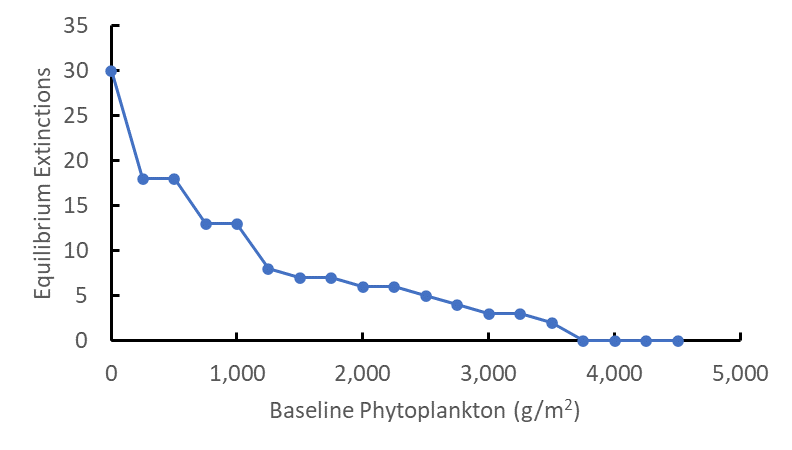


**Supplementary Figure S8** – Steady-state biomass values of six trophic categories – foodweb phytoplankton, algae, filter feeders, herbivores, omnivores, and top predators – as baseline phytoplankton was varied upward (compare with Supplementary Figure S7). As baseline phytoplankton increased, algae biomass was suppressed by the model’s shared carrying capacity. Filter feeder biomass followed a similar trend to phytoplankton biomass. Herbivore biomass followed a similar trend to algal biomass. Biomass of omnivores and predators increased with increasing baseline phytoplankton owing to their reliance on filter feeder prey relative to herbivore prey (compare absolute biomass between filter feeder and herbivore species).


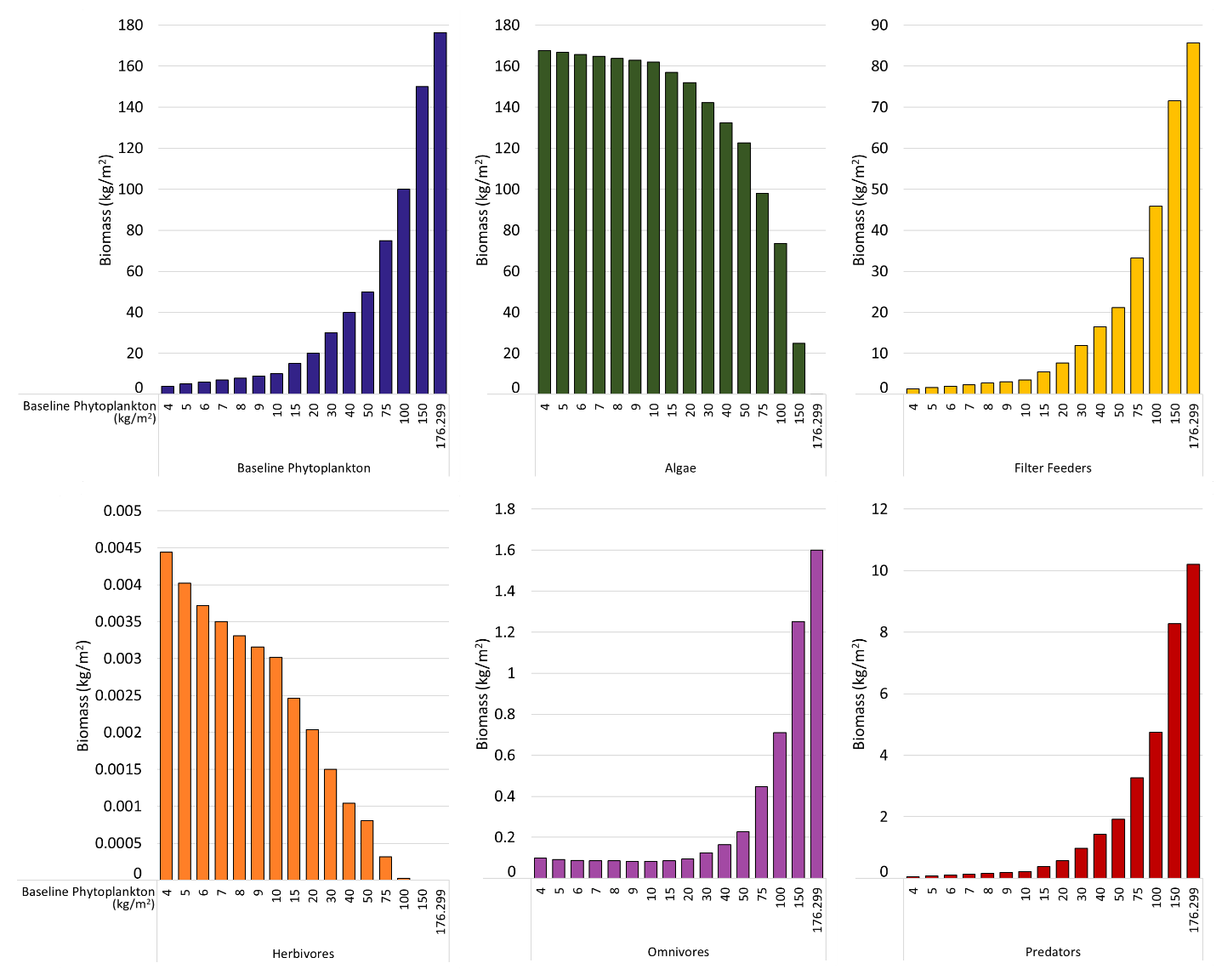


**Supplementary Figure S9** – Simulation strategy highlighting ‘equilibration period’ followed by ‘treatment period’. (A) Baseline phytoplankton is active for one year of simulation time with food web phytoplankton and offshore phytoplankton deactivated. Offshore phytoplankton is then activated and one year of empirical offshore phytoplankton data is simulated. (B) The resulting biomass curves of all 105 non-phytoplankton species.


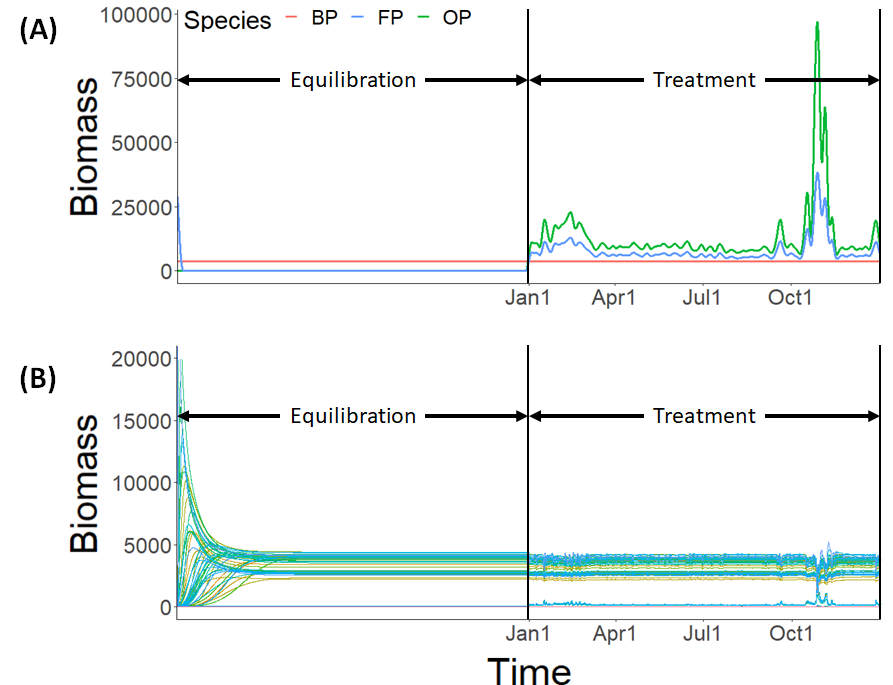

Supplement: Supplementary file 1 — Supplementary Information. [file 41598_2024_57108_MOESM1_ESM.docx]
